# Mutational Landscape and Clinical Impact of SPEN Mutations in Patients with Chronic Lymphocytic Leukemia

**DOI:** 10.3390/cancers17213586

**Published:** 2025-11-06

**Authors:** Priyatharsini Nirmalanantham, Andrés E. Quesada, Anindita Ghosh, Pei Lin, Chi Y. Ok, Richard K. Yang, Hong Fang, Sofia Garces, Rashmi Kanagal-Shamanna, Sanam Loghavi, Mark J. Routbort, Cameron Cheng Yin, Wang Wei, Sarah Pasyar, Roland Bassett, Siba El Hussein, Nitin Jain, Jan Burger, William G. Wierda, Sa Wang, Carlos Bueso-Ramos, Keyur P. Patel, Leonard Jeffrey Medeiros, Fatima Zahra Jelloul

**Affiliations:** 1 Department of Hematopathology, The University of Texas MD Anderson Cancer Center, Houston, TX 77030, USA; nirmalanantham.p@outlook.com (P.N.); aquesada@mdanderson.org (A.E.Q.); aninditagh@yahoo.com (A.G.); hfang@mdanderson.org (H.F.); cyin@mdanderson.org (C.C.Y.);; 2 Department of Biostatistics, The University of Texas MD Anderson Cancer Center, Houston, TX 77030, USA; 3 Department Pathology, The University of Vermont Medical Center, Burlington, VT 05401, USA; 4 Department of Leukemia, The University of Texas MD Anderson Cancer Center, Houston, TX 77030, USA

**Keywords:** chronic lymphocytic leukemia, SPEN mutations, NOTCH1 regulatory pathway

## Abstract

**Simple Summary:**

*SPEN* mutations have been described in a limited number of CLL cases and previous studies have suggested that mutations in the NOTCH1 regulatory pathway including *SPEN* are associated with adverse patient outcomes. However, the clinicopathologic features including the molecular landscape of *SPEN* mutations in CLL patients have not been extensively studied. We conducted this study to assess the frequency of *SPEN* mutations and their potential clinical impact on outcomes in a large cohort of CLL patients. We also describe co-occurring gene alterations in this cohort. We show in this study that *SPEN* mutations are associated with shorter time-to-first treatment in CLL patients.

**Abstract:**

Background/Objectives: *NOTCH1* is frequently mutated in chronic lymphocytic leukemia (CLL) and is a marker of poor prognosis. In addition to *NOTCH1*, mutations in the NOTCH1 regulatory pathway including *SPEN* have been described in a limited number of CLL cases and others have suggested that these mutations are also associated with adverse patient outcomes Methods: In this study, 1617 CLL cases were assessed using targeted sequencing and a 29-gene panel and the results were correlated with prognosis. Results: *SPEN* mutations were detected in 48 (2.9%) CLL patients: 92.4% were deleterious (frameshift or truncating nonsense mutations) and the remaining (7.6%) were missense. Compared with *SPEN* wild type CLL patients, *SPEN* mutated patients had a statistically higher frequency of IGHV unmutated status (79.5% vs. 57.8%, *p* = 0.004), CD38 positivity (73.3% vs. 52.4%, *p* = 0.01), ZAP70 positivity (77.3% vs. 58.3%, *p* = 0.01) and trisomy 12 (43.5% vs. 13.7%, *p* < 0.001). The most common gene mutations co-occurring with *SPEN* mutations were as follows: *NOTCH1* (43.7%), *TP53* (22.9%), *BIRC3* (12.5%), *SF3B1* (10.4%), *XPO1* (8.3%), *MUC2* (6.2%), *ATM* (4.2%), *FBXW7* (4.2%), and *BTK* (4.2%). Patients with *SPEN* mutated CLL had a significantly shorter time-to-first treatment compared to CLL patients with wild type *SPEN* (2.5 vs. 4.07 years, *p* = 0.01). The finding of shorter time-to-first treatment in *SPEN* mutated CLL patients was not maintained in a multivariable analysis. IGHV unmutated status, *TP53* disruption, and trisomy 12 remained independently predictive of a shorter time-to-first treatment in a multivariable analysis. Conclusions: These data show that *SPEN* mutations in CLL are associated with adverse prognostic impact and should be included in sequencing assays performed for the prognostic workup of CLL patients.

## 1. Introduction

Chronic lymphocytic leukemia (CLL) is the most frequent leukemia affecting adults in the western world [1,2]. Despite increased understanding of CLL pathogenesis and the introduction of new therapies, CLL remains an incurable disease. CLL patients can have diverse clinical courses: some patients have an indolent course whereas others require therapy shortly after diagnosis. Many clinicopathologic features have been shown to have value in predicting prognosis, including clinical stage, expression of CD38 and/or ZAP70, somatic mutations of the immunoglobulin heavy chain variable (IGHV) genes, identification of chromosomal abnormalities by conventional cytogenetics or fluorescence in situ hybridization, including deletions of chromosome loci 13q14, 17p13, and 11q23, and trisomy 12 [3,4,5,6]. More recently, in depth genomic characterization of CLL cases has shown great heterogeneity at the molecular level. A number of genes are recurrently mutated in CLL, and, in particular, *TP53*, *NOTCH1*, and *SF3B1* are associated with adverse clinical outcomes [7,8,9,10,11].

NOTCH1 is a cell surface receptor that releases its intracellular domain as a transcription factor upon activation, and plays key roles in cell proliferation, differentiation, and apoptosis. After binding to its ligand, NOTCH1 undergoes two successive proteolytic cleavages resulting in the release of its active form, which translocates into the nucleus, thereby mediating transcriptional activation of target genes including *TP53*, *MYC*, and components of the nuclear factor kB (NF-kB) pathway. NOTCH1 signaling has been shown to be important in CLL pathogenesis and is one of the most frequently mutated genes associated with advanced stage at diagnosis and poorer overall survival [9].

Several proteins negatively regulate NOTCH1 activity, including the tumor repressors F-box/WD repeat-containing protein 7 (FBXW7), mediator complex subunit 12 (MED12), and SPEN family transcriptional repressor (SPEN) [9,11,12]. Whereas FBXW7 and MED12 act to prevent proteasomal degradation of NOTCH1, SPEN directly interferes with NOTCH1 signaling. SPEN encodes for an adaptor protein that is a part of the Mint/SHARP/SPEN complex. This complex interacts with several transcription factors, including Msx2 and RBP-J, and acts as a bridge between RBP-J and repressor proteins, such as NCor/SMRT/HDACs. RBPJ is essential for DNA binding of the NOTCH1 intracellular domain (NICD1). SPEN functions as a co-repressor of RBPJ, the nuclear effector of the Notch pathway [9,11,12]. Mutations impacting the function of *FBXW7*, *MED12*, and *SPEN*, by inactivating their repressor function, result in enhanced NOTCH1 signaling which may influence patient outcomes via NOTCH1 activation.

Inactivating *SPEN* mutations have been reported in a limited number of CLL cases assessed with a frequency ranging from approximately 1% to 9% [12,13,14,15]. Others have reported that mutations in the NOTCH1 regulatory pathway including *SPEN* predict a shorter time-to-first treatment in a small cohort of CLL patients [15]. We conducted this study to assess the frequency of SPEN mutations and their potential clinical impact on outcomes in a large cohort of CLL patients. We also describe co-occurring gene alterations in this cohort.

## 2. Materials and Methods

### 2.1. Patients and Samples

Samples of peripheral blood and bone marrow aspirates from 1617 CLL patients were subjected to targeted sequencing using a 29-gene panel (EndCLL Assay V1). The percentage of CLL cells in each sample was estimated by multi-color flow cytometry immunophenotypic analysis. Only samples that contained at least 10% monotypic CD5+/CD19+ B cells underwent sequencing with the EndCLL Assay V1.

### 2.2. Clinical and Laboratory Characteristics

Demographic, clinical, and laboratory data included age, gender, prior treatments, clinical follow-up, flow cytometry immunophenotypic findings, expression of ZAP70 and CD38, IGHV mutation status, and fluorescence in situ hybridization (FISH) results. These data were evaluated and collected at the time (±60 days) of next generation sequencing sample collection. Static prognostic factors, such as *IGHV* mutation status and ZAP70 expression, were not repeated at time of sample collection for NGS if already known. These data were extracted from the medical records using an Institutional Review Board-approved chart review protocol.

### 2.3. Targeted Next Generation Gene Sequencing

The gene panel employed included 29 genes known to be mutated in CLL as has been described in [16]. This panel included the following: *ATM*, *BIRC3*, *BTK*, *CALR*, *CARD11*, *CD79A*, *CD79B*, *CHD2*, *CSMD3*, *CXCR4*, *DDX3X*, *EZH2*, *FAT1*, *FBXW7*, *KLHL6*, *LRP1B*, *MAPK1*, *MUC2*, *MYD88*, *NOTCH1*, *PLCG2*, *PLEKHG5*, *POT1*, *SF3B1*, *SPEN*, *TGM7*, *TP53*, *XPO1*, and *ZMYM3*. Briefly, sequencing libraries for target regions in 29 genes were prepared from 250 ng of genomic DNA isolated from peripheral blood or bone marrow samples. The Agilent Haloplex HS target enrichment system (Agilent Technologies, Santa Clara, CA, USA) was utilized for the library preparation. Incorporation of molecular barcodes in the DNA library allowed removal of duplicate reads and improved sensitivity of detection at low variant allelic frequencies (VAF), a useful design feature suitable for the detection of subclonal mutations in CLL. Paired-end bidirectional sequencing was performed using the Illumina MiSeq platform (Illumina Inc., San Diego, CA, USA). A minimum average depth of 3000× per sample, and a minimum of 80% reads at a quality score of AQ30 or better were required for the interpretation. Paired germline testing was not performed due to challenges of obtaining a reliable germline sample (i.e., cultured skin fibroblasts) for routine testing in hematological malignancies. Variant calling was performed using Agilent SureCall software 4.2.2 (Agilent Technologies) and for variants not listed in single nucleotide polymorphism databases, we took into account the tumor cell percentage, cytogenetics data for chromosomal changes, VAF of novel variant, and VAF of known somatic mutation to identify variants of potential germline variants. An internal database of all the variants along with the distribution of VAF to identify potential germline variants was employed. Variants with heterozygous or homozygous VAF without supporting literature confirming somatic origin or without the tumor percentage and VAF data supporting somatic origin were reported separately from the known somatic mutations as variants of uncertain origin and removed from the final analysis/cohort. Final review and reporting of the results were performed by a pathologist.

### 2.4. Statistical Analyses

The patient baseline characteristics were summarized using descriptive statistics. Wilcoxon rank-sum and Chi-square tests were used to assess the association between SPEN mutational status and continuous and categorical variables, respectively. The Kaplan–Meier method was used to estimate the time to starting first treatment from the diagnosis date and the log-rank test was used to compare the different groups. All statistical analyses were performed using SAS version 9.4. All statistical tests used a significance level of 5%. The association between time-to-first treatment and known risk factors were evaluated using univariate and multivariate Cox proportional hazard models.

## 3. Results

### 3.1. Patients and Clinical Characteristics

The CLL patients included in this study are summarized in Table 1, according to *SPEN* mutational status. Overall, we detected *SPEN* mutations in 48 of 1617 (2.9%) CLL cases. The median age of patients at diagnosis of *SPEN* mutated CLL was 65 years (range, 40–87) with a male to female ratio of 1.3:1. There were 31 (64.6%) treatment-naïve and 17 (35.4%) previously treated patients (relapsed/refractory). At the time of data collection, 8 (16.7%) patients had died. The median follow-up interval was 9.9 years.

### 3.2. SPEN Mutations

**Figure 1:**
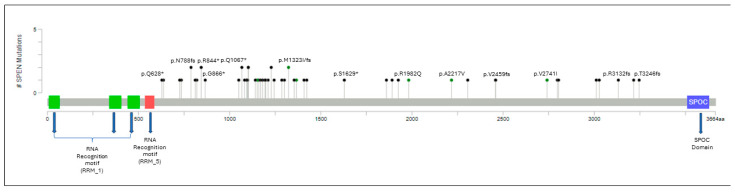
Lollipop plot illustrating the distribution of SPEN mutations along the gene. Circles are colored according to the corresponding mutation type. Missense mutations are shown in green circles, and truncating mutations, including nonsense and frameshift mutations, are shown in black circles. At a given locus, lollipop height is proportional to the number of times a mutation was observed in the study subjects. The structural domains of the gene are represented in the figure: RRM: RNA recognition motifs that confer binding to a steroid receptor RNA coactivator. SPOC: carboxy-terminal domain that permits binding to other corepressor proteins. *SPEN* mutations are located toward the carboxy-terminal, upstream of the SPOC domain.

A total of 53 individual *SPEN* mutations were identified in 48 CLL cases. A detailed summary of these mutations is shown in Table 2, Figure 1 and Supplementary Table S1. In 10 (21%) patients, >1 *SPEN* mutation was detected. A total of 49 of 53 (92.4%) *SPEN* mutations were deleterious (frameshift or truncating nonsense mutations) with the remaining 4 (7.6%) mutations being missense. *SPEN* mutations were located toward the carboxy-terminal, upstream of the SPEN paralog and ortholog C-terminal (SPOC) domain.

### 3.3. Concurrent Gene Mutations

**Figure 2:**
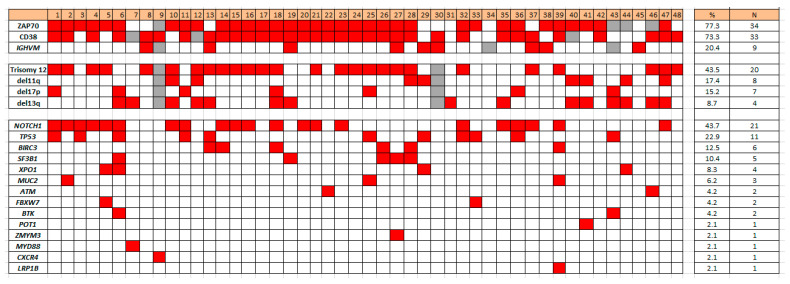
Heat map illustrating co-occurring mutations and other biomarkers in *SPEN* mutated CLL.

Concurrent gene mutations detected in the 48 CLL cases with *SPEN* mutations included the following: 21 (43.7%) *NOTCH1*, 11 (22.9%) *TP53*, 6 (12.5%) *BIRC3*, 5 (10.4%) *SF3B1*, 4 (8.3%) *XPO1*, 3 (6.2%) *MUC2*, 2 (4.2%) *ATM*, 2 (4.2%) *FBXW7*, 2 (4.2%) *BTK*, 1 (2.1%) *POT1*, 1 (2.1%) *ZMYM3*, 1 (2.1%) *MYD88*, 1 (2.1%) *CXCR4*, and 1 (2.1%) *LRP1B* (Figure 1). *FAT1*, *DDX3X*, *CALR*, *PLCG2*, and *CARD11* were not mutated in cases of CLL with *SPEN* mutation (Figure 2).

### 3.4. Correlation with Other CLL Prognostic Biomarkers

Compared with CLL patients with *SPEN* wild type, CLL patients with *SPEN* mutations had a statistically higher frequency of *IGHV* unmutated status (79.5% vs. 57.8%, *p* = 0.004), CD38 positivity (73.3% vs. 52.4%, *p* = 0.01), ZAP70 expression (77.3% vs. 58.3%, *p* = 0.01), and trisomy 12 (43.5% vs. 13.7%, *p* < 0.001).

### 3.5. SPEN Mutations Carry Independent Prognostic Impact in CLL Patients

**Figure 3:**
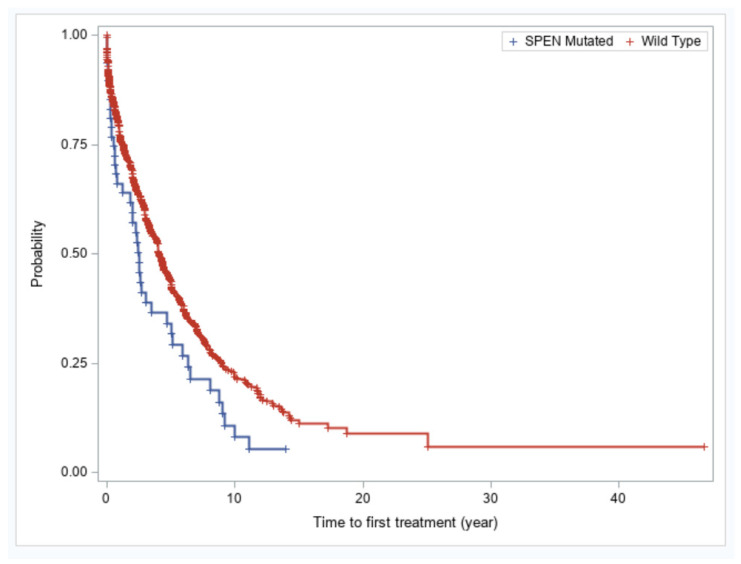
Kaplan–Meier plots of showing time-to-first treatment for patients with *SPEN* mutated versus *SPEN* wild type CLL.

We evaluated whether *SPEN* mutations in CLL confer an adverse prognosis. Patients with CLL with *SPEN* mutations had significantly shorter time-to-first treatment compared with patients with wild type *SPEN* CLL (2.5 vs. 4.07 years, *p* = 0.01) (Figure 3). The finding of shorter time-to-first treatment in *SPEN* mutated CLL patients was not maintained in a multivariable analysis. *IGHV* unmutated status, *TP53* disruption, and trisomy 12 remained independently predictive of a shorter time-to-first treatment in a multivariable analysis (Table 3).

## 4. Discussion

Several genes encoding proteins that regulate the NOTCH signaling pathway have been shown to be recurrently mutated in CLL. In addition to activating *NOTCH1* mutations, which prolong NICD1 transcription factor activity [17], other genomic alterations have been shown to interfere with NOTCH1 signaling. These alterations include non-coding *NOTCH1* mutations in the 3′ untranslated region [17], *SPEN* mutations [12,13,14,15,18], *FBXW7* mutations [13,14,15,18,19], *MED12* mutations [11,13,18], and probably alterations of the transcription factor RBPJ [12]. *SPEN* mutations have been described in a limited number of CLL cases in patients with treatment-naïve and relapsed/refractory disease [12,13,14,15,18]. However, the clinicopathologic features including the molecular landscape of *SPEN* mutations have not been extensively studied. In the current study, we assessed a large cohort of CLL patients for *SPEN* mutations. We identified deleterious *SPEN* mutations in approximately 3% of CLL cases in this cohort. The most frequent mutation co-occurring with *SPEN* mutation in this cohort was *NOTCH1*, in about half of cases. Other mutations that occurred in *SPEN* mutated CLL included *TP53*, *BIRC3*, *SF3B1*, *XPO1*, *MUC2*, *FBXW7*, *ATM*, *POT1*, *MYD88*, *CXCR4*, and *ZMYM3*.

Others have reported NOTCH1 regulatory pathway mutations in a small cohort of CLL patients [15], including five patients with *SPEN* mutations, and found that these patients had a significantly higher frequency of *IGHV* unmutated, CD38 positivity, ZAP70 positivity, deletion 11q, and trisomy 12. We also show in this cohort that *SPEN* mutations in CLL are significantly associated with unmutated *IGHV*, CD38 positivity, ZAP70 expression, and trisomy 12.

Others have shown that coding and non-coding *NOTCH1* mutations negatively impact prognosis in CLL patients [17,18,20,21]. NOTCH1 activation is also associated with aggressive CLL clinical behavior and decreased time-to-first treatment [22,23], but this association does not correlate with *NOTCH1* and *IGHV* mutational status [22]. We establish in this study that mutations of *SPEN*, a gene in the NOTCH1 regulatory pathway, are associated with shorter time-to-first treatment. Further studies are needed to determine whether *SPEN* mutations as well as other NOTCH1 regulatory pathway mutations are associated with increased transformation risk or poor response to frontline chemo-immunotherapy or targeted agents currently used for CLL patients.

NOTCH1 has a critical pathogenic role in CLL. In addition to *NOTCH1* mutations, approximately half of CLL cases devoid of mutations express the active form of NOTCH1 ICN1 (intracellular portion of NOTCH1), thus implicating a much broader role for this transcription factor in CLL pathogenesis [22,23]. NOTCH1 target genes include key regulators of B-cell proliferation, survival, and signal transduction. NOTCH1 is known to transactivate MYC via binding to B-cell-specific regulatory elements, thus implicating this oncogene in CLL development. The results of this study substantially extend the role of NOTCH1 in CLL pathogenesis and have direct implications for specific therapeutic targeting.

Current International Working Group (IW) CLL guidelines recommend mutational sequencing for *TP53* [24]. However, many current clinically available mutation panels sequence additional genes and provide information of undefined clinical impact. In addition, based on the adverse prognostic significance of *TP53*, *NOTCH1*, *BIRC3*, and *SF3B1* mutations in CLL [25,26,27,28,29], Rossi et al. integrated mutations/disruptions into a cytogenetic model that independently predicts survival and improves prognostication of CLL patients [30]. We suggest that the presence of *SPEN* mutation may add to the currently available prognostic models.

We acknowledge the limitations of this study. Our institution is a referral center and there may be selection bias. In addition, not all patients in this cohort underwent gene mutational testing at the time of CLL diagnosis. It is therefore possible that some *SPEN* mutations were not present at initial diagnosis and were acquired because of clonal evolution. However, we believe that later acquisition of *SPEN* mutations would be unlikely as the median time from diagnosis to next generation sequencing was approximately 3 years in our cohort.

## 5. Conclusions

In summary, we show that *SPEN* mutations have a prognostic impact in CLL patients. We also show that *SPEN* mutations frequently co-occur with *NOTCH1* mutations and are associated with similar baseline factors to *NOTCH1* mutated CLL, such as *IGHV* unmutated status, CD38 positivity, ZAP70 expression, and trisomy 12. Most importantly, the findings we present emphasize the importance of screening for *SPEN* mutations as their detection may help to identify CLL patients with an adverse prognosis.

## Figures and Tables

**Table 1:** Characteristics of CLL patients according to *SPEN* mutation status.

	SPEN M48.	Scheme 1569.	*p* = Value
**Characteristics**	N (%)	N (%)	
Age at diagnosis (year)	65 (40–87)	64 (36–89)	0.20
Gender			
Female	21 (44)	318 (32)	0.10
Male	27 (56)	665 (67)	
Ratio (M:F)	27/21	665/318	
Unknown	0	586	
Treatment-naïve	31 (64.6)	166 (62.6)	0.80
Relapse/Refractory	17 (35.4)	99 (37.3)	
Unknown	0	1304	
Time-to-first treatment (years)	2.5	4.07	0.01
IGHV			
IGHV unmutated	35 (79.5)	435 (57.8)	0.004
IGHV mutated	9 (20.4)	317 (42.1)	
Unknown	4	817	
ZAP70			
ZAP70 positive	34 (77.3)	452 (58.3)	0.01
ZAP70 negative	10 (22.7)	323 (41.7)	
ZAP70 unknown	4	794	
CD38			
CD38 positive	33 (73.3)	436 (52.4)	0.01
CD38 negative	12 (26.7)	396 (47.6)	
CD38 unknown	3	737	
CG Hierarchical Model			
Del17p	7 (15.2%)	93 (16.5%)	0.80
Del11q	8 (17.4%)	96 (17.1%)	0.90
Trisomy 12	20 (43.5%)	77 (13.7%)	<0.001
Del13q	4 (8.7%)	210 (37.4%)	<0.001
0	7 (15.2%)	86 (15.3%)	0.90
Unknown	2	1007	

**Table 2:** Genetic variants and frequencies of *SPEN* mutations.

Genomic Position	Protein Change	Number of Patients	%
c.3420_3421dupA	p.P1141fs	1	2.1
c.1882C > T	p.Q628*	1	2.1
1909C > T	p.R637*	1	2.1
c.3290_3301delinsCTGATTT	p.E1097fs	1	2.1
c.3308C > A	p.S1103*	2	4.2
c.2363dupA	p.N788fs	2	4.2
c.3967_3968del	p.M1323fs	1	2.1
c.2431_2432del	p.K811fs	1	2.1
c.2530C > T	p.R844*	2	4.2
c.2596G > T	p.G866*	1	2.1
c.9079C > T	p.R3027*	1	2.1
c.3150_3154del	p.K1050fs	1	2.1
c.3199C > T	p.Q1067*	2	4.2
c.3245C > G	p.S1082*	1	2.1
c.2460_2500del	p.D820fs	1	2.1
c.9645_9646dupGG	p.V3216fs	1	2.1
c.3276del	p.G1093fs	1	2.1
c.3295C > T	p.Q1099*	1	2.1
c.3304C > T	p.Q1102*	2	4.2
c.3452A > G	p.H1151R	1	2.1
c.3477dupT	p.G1160fs	1	2.1
c.3508C > T	p.R1170*	1	2.1
c.3541C > T	p.Q1181*	1	2.1
c.3581del	p.S1194fs	1	2.1
c.3591dupA	p.D1198fs	1	2.1
c.3632_3654del	p.V1211fs	1	2.1
c.8406_8407del	p.A2804fs	1	2.1
c.3682_3686del	p.K1228fs	1	2.1
c.3682A > T	p.K1228*	1	2.1
c.3732_3736del	p.N1244fs	1	2.1
c.3855_3867del	p.G1286*	1	2.1
c.3900_3903del	p.I1300fs	1	2.1
c.3969G > A	p.M1323I	1	2.1
c.4060_4061del	p.S1354fs	1	2.1
c.4097G > A	p.R1366Q	1	2.1
c.4223T > A	p.L1408*	1	2.1
c.4268_4269del	p.S1423*	1	2.1
c.4886C > G	p.S1629*	1	2.1
c.5578G > T	p.G1860*	1	2.1
c.2176C > T	p.Q726*	1	2.1
c.5671G > T	p.E1891*	1	2.1
c.5773G > T	p.E1925*	1	2.1
c.5945G > A	p.R1982Q	1	2.1
c.6650C > T	p.A2217V	1	2.1
c.6915_6917del	p.N2306fs	1	2.1
c.7373del	p.D2458fs	1	2.1
c.7375_7381delins8	p.V2459fs	1	2.1
c.8221G > A	p.V2741I	1	2.1
c.8388_8391del	p.P2797fs	1	2.1
c.9031C > T	p.R3011*	1	2.1
c.9394_9395del	p.R3132fs	1	2.1
c.2203C > T	p.Q735*	1	2.1
c.9731_9737del	p.T3246fs	1	2.1

**Table 3:** Univariate and multivariable analyses of Cox proportional hazard analysis of time-to-first treatment in CLL cohort.

	Univariate		Multivariate	
	Hazard Ratio (95% CI)	*p* Values	Hazard Ratio (95% CI)	*p* Values
Age at CLL diagnosis	1.00 (0.99–1.00)	0.55	0.99 (0.98–1.00)	0.15
Sex Male: Female	1.03 (0.87–1.22)	0.72	1.10 (0.85–1.42)	0.48
IGHV unmutated status	0.37 (0.30–0.46)	<0.001	0.44 (0.32–0.63)	<0.001
ZAP70 expression	2.03 (1.67–2.48)	<0.001	1.23 (0.89–1.67)	0.21
*TP53* disruption	1.69 (1.35–2.13)	<0.001	1.87 (1.25–2.82)	0.003
Del(13q)	0.47 (0.36–0.60)	<0.001	0.93 (0.58–1.49)	0.76
Trisomy 12	1.30 (0.97–1.74)	0.08	1.90 (1.09–3.34)	0.02
Del11q	1.77 (1.34–2.33)	<0.001	1.45 (0.85–2.48)	0.17
*SPEN* mutated	1.47 (1.07–2.02)	0.02	1.26 (0.85–1.88)	0.25
*NOTCH1* mutated	1.50 (1.25–1.80)	<0.001	0.77 (0.55–1.08)	0.13

## Data Availability

Data presented in this study are available upon request from the corresponding author.

## Supplementary Materials

The following supporting information can be downloaded at: https://www.mdpi.com/article/10.3390/cancers17213586/s1, Supplementary Table S1: Variant Allelic frequencies of SPEN Mutations.
