# Supplementary material for: Mutational Landscape and Clinical Impact of SPEN Mutations in Patients with Chronic Lymphocytic Leukemia
Source: Cancers (Basel). 2025 Nov 6;17(21):3586. doi: 10.3390/cancers17213586 (PMC12607580; doi:10.3390/cancers17213586)
Supplement: Supplementary file 1 [file cancers-17-03586-s001.zip › cancers-3907871-supplementary.pdf]

Supplementary Table 1: Variant Allelic frequencies of *SPEN* Mutations.

Genomic Position	Protein Change	VAF (%)
c.3420_3421dupA	p.P1141fs	7
c.1882C>T	p.Q628*	31
c.1909C>T	p.R637*	<5%
c.3290_3301delinsCTGATTT	p.E1097fs	23
c.3308C>A	p.S1103*	39
c.3308C>A	p.S1103*	<5%
c.2363dupA	p.N788fs	20
c.2363dupA	p.N788fs	<5%
c.3967_3968del	p.M1323fs	<5%
c.2431_2432del	p.K811fs	47
c.2530C>T	p.R844*	27
c.2530C>T	p.R844*	74
c.2596G>T	p.G866*	5
c.9079C>T	p.R3027*	<5%
c.3150_3154del	p.K1050fs	11
c.3199C>T	p.Q1067*	<5%
c.3245C>G	p.S1082*	23
c.2460_2500del	p.D820fs	30
c.9645_9646dupGG	p.V3216fs	9
c.3276del	p.G1093fs	<5
c.3295C>T	p.Q1099*	6
c.3304C>T	p.Q1102*	75
c.3304C>T	p.Q1102*	91
c.3452A>G	p.H1151R	50
c.3477dupT	p.G1160fs	<5%
c.3508C>T	p.R1170*	23
c.3541C>T	p.Q1181*	40
c.3581del	p.S1194fs	21
c.3591dupA	p.D1198fs	65
c.3632_3654del	p.V1211fs	25
c.8406_8407del	p.A2804fs	42
c.3682_3686del	p.K1228fs	<5
c.3682A>T	p.K1228*	14
c.3732_3736del	p.N1244fs	<5
c.3855_3867del	p.G1286*	6
c.3900_3903del	p.I1300fs	<5
c.3969G>A	p.M1323I	24

c.4060_4061del	p.S1354fs	<5
c.4097G>A	p.R1366Q	21
c.4223T>A	p.L1408*	11
c.4268_4269del	p.S1423*	5
c.4886C>G	p.S1629*	44
c.5578G>T	p.G1860*	7
c.2176C>T	p.Q726*	<5
c.5671G>T	p.E1891*	<5
c.5773G>T	p.E1925*	<5
c.5945G>A	p.R1982Q	<5
c.6650C>T	p.A2217V	49
c.6915_6917del	p.N2306fs	45
c.7373del	p.D2458fs	16
c.7374_7375insT	p.V2459fs	<5
c.8221G>A	p.V2741I	36
c.8388_8391del	p.P2797fs	<5
c.9031C>T	p.R3011*	<5
c.9394_9395del	p.R3132fs	<5
c.2203C>T	p.Q735*	<5
c.9731_9737del	p.T3246fs	84
